# Supplementary material for: Energy-resolved EBSD using a monolithic direct electron detector
Source: Ultramicroscopy. Author manuscript; Available in PMC 2026 Jul 18. (PMC13379735; doi:10.1016/j.ultramic.2025.114301)
Supplement: 1 [file NIHMS2188400-supplement-1.pdf]

Supplementary Information of

Energy-Resolved EBSD using a Monolithic Direct Electron Detector

by

N. M. Della Ventura, K. Moore
et al.

Materials Department, University of California, Santa Barbara, Santa Barbara, CA 93106, USA
Direct Electron L.P., San Diego, CA, USA
Department of Materials Science and Engineering, Carnegie Mellon University, 5000 Forbes Avenue, Pittsburgh PA 15213, USA

S.1 - Calculation of the width of a Kikuchi band extending vertically across the detector

In the following, we report the equation employed to determine the width, w, of a Kikuchi band that extends vertically across the detector plane. Specifically, w, expressed in pixel, can be calculated as:

$$w = \frac{1}{\delta} \cdot \frac{\lambda}{d} \cdot \sqrt{L^2 + \left((y_{\mathrm{PC}} - y) \cdot \delta\right)^2} \tag{1}$$

with:

$$\delta = p_s \cdot b, \quad \lambda = \frac{h}{\sqrt{2m\,|e|\,V}}, \quad d_{hkl,\,\mathrm{FCC}} = \frac{a}{\sqrt{h^2 + k^2 + l^2}}, \quad L = N_y \cdot \delta \cdot z^*, \quad y_{\mathrm{PC}} = N_y \cdot (1 - y^*), \quad x_{\mathrm{PC}} = N_x \cdot x^*$$

In the equations above, p_s is the physical size of every pixel constituting the detector and is given in micrometers, b is the binning factor, λ denotes the non-relativistic de Broglie wavelength in meters, $h = 6.626 \times 10^{-34}$ J·s is the Planck's constant, $m = 9.109 \times 10^{-31}$ kg represents the electron mass, $|e| = 1.60217 \times 10^{-19}$ C is the electron charge, V indicates the voltage of the primary beam in volts (not keV), d represents the interplanar spacing of the plane with Miller indices (hkl), a is the lattice parameter in meters, L denotes the camera length, N_x and N_y are the detector sizes along x and y expressed in pixels upon binning (for our detector $N_x = N_y$), and (x^*, y^*, z^*) indicate the coordinates of the pattern center (PC). Note that also $(x_{\mathrm{PC}}, y_{\mathrm{PC}})$ are the PC coordinates, however, $(x_{\mathrm{PC}}, y_{\mathrm{PC}})$ are expressed in pixels and go from left to right of the detector (for x_{PC}) and from top to bottom of the detector (for y_{PC}), respectively, whilst (x^*, y^*) are expressed in fraction of the detector size and go from left to right of the detector (for x^*) and from bottom to top of the detector (for y^*), respectively (see Figure S.1).The variable y in Equation 1 identifies the vertical detector coordinate (in pixels, from top to bottom of the detector) at which the Kikuchi band width is determined.

For this study, p_s (2,048 × 2,048 px^2 detector) = 13 µm, $b = 8$, V = 12,000 V, $a_{\mathrm{Si}} = 0.543 \times 10^{-9}$ m, $N_x = N_y$ = 256 px, and (x^*, y^*, z^*) = (0.4496, 0.5512, 1.3495). Therefore, for y_1 = 49 px and y_2 = 225 px, w_1 = 20.51 px and w_2 = 21.15 px, respectively.

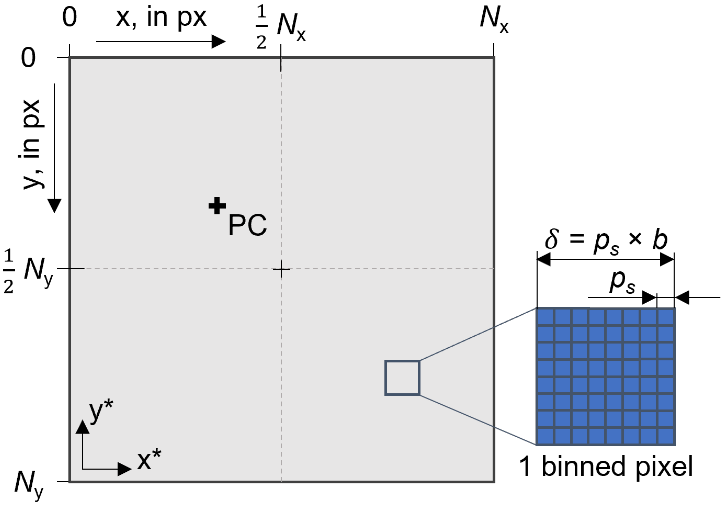

Figure S.1: Schematic of the detector illustrating some of the parameters used in Equation 1 to calculate the width of the Kikuchi band that extends vertically across the detector plane.

Supplementary Figures of

Energy-Resolved EBSD using a Monolithic Direct Electron Detector

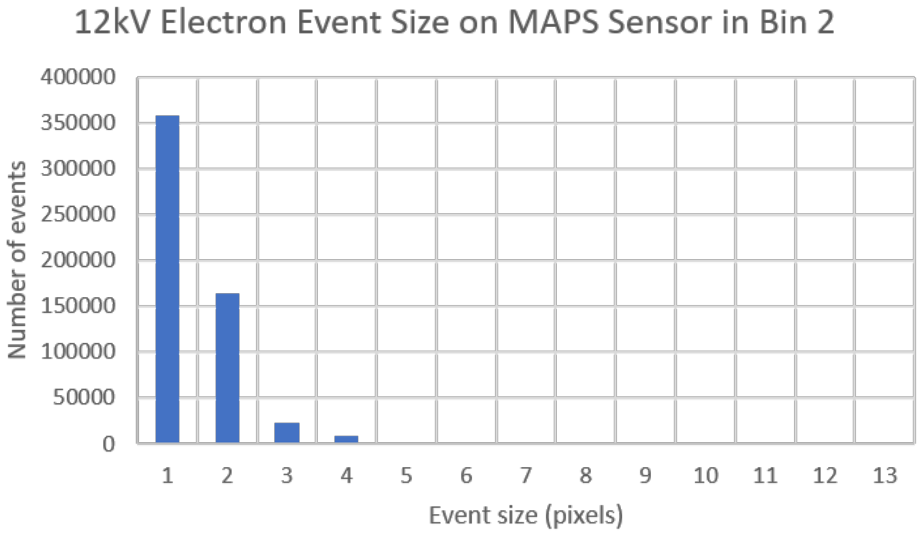

Figure S.2: Graph of electron event sizes for 12 keV on the DE-SEMCam.The average event size is 1.4 pixels in 2x binning mode (2,048 × 2,048 pixels).

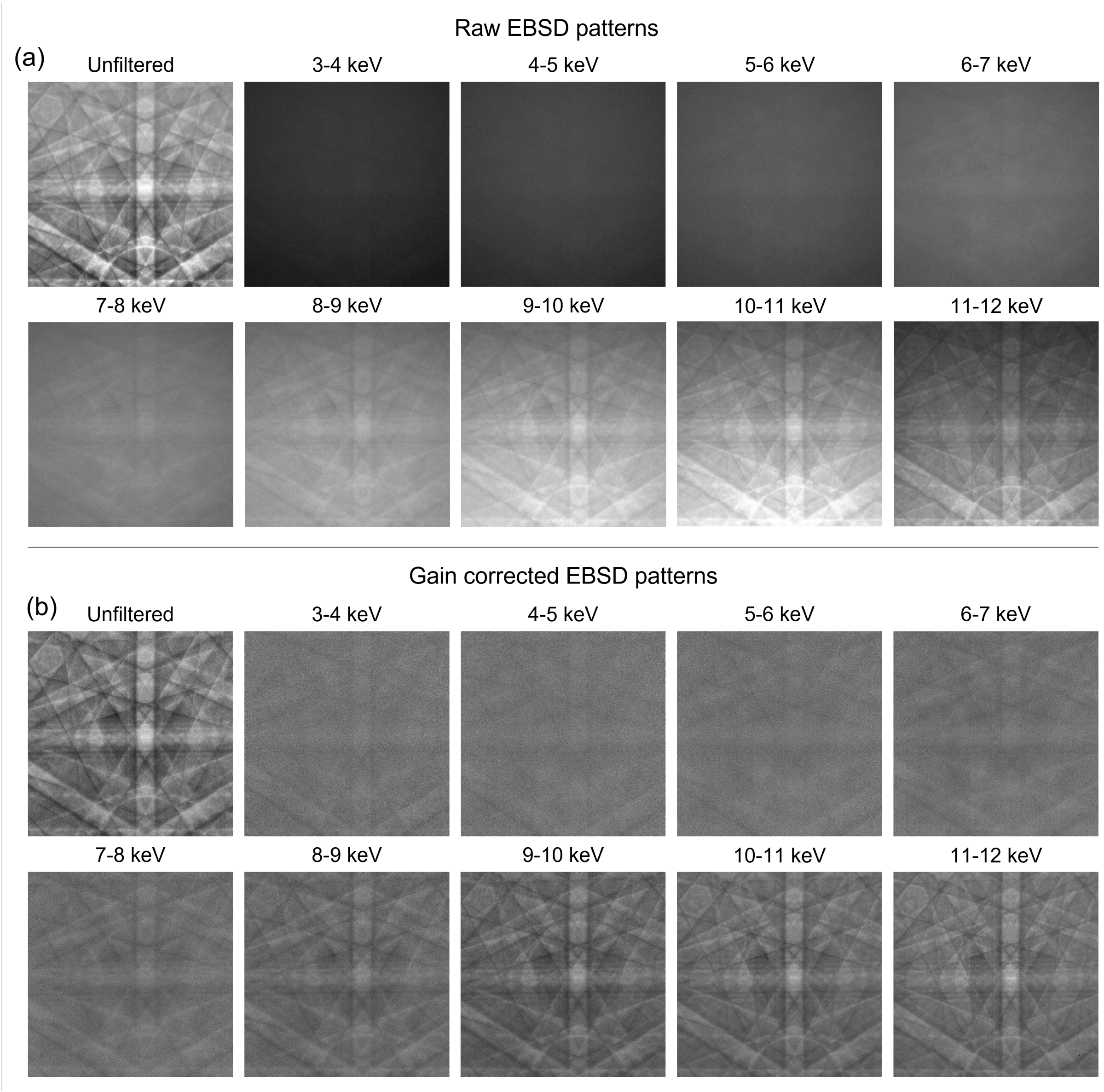

Figure S.3: (a) Full set of 1 keV-wide energy-filtered EBSD patterns reconstructed from the 3–12 keV range, with all energy-filtered patterns normalized to a common intensity scale (excluding the unfiltered reference). (b) Corresponding gain-corrected energy-filtered EBSD patterns, likewise normalized to a common scale, except for the unfiltered pattern.
